# Reformulating and Mirroring in Psychotherapy: A Conversation Analytic Perspective

**DOI:** 10.3389/fpsyg.2020.00318

**Published:** 2020-03-05

**Authors:** A. S. L. Knol, Mike Huiskes, Tom Koole, Reitske Meganck, Tom Loeys, Mattias Desmet

**Affiliations:** ^1^Department of Psychoanalysis and Clinical Consulting, Ghent University, Ghent, Belgium; ^2^Center for Language and Cognition Groningen, University of Groningen, Groningen, Netherlands; ^3^Health Communication Research Unit, University of the Witwatersrand, Johannesburg, South Africa; ^4^Department of Data-analysis, Ghent University, Ghent, Belgium

**Keywords:** psychodynamic therapy, cognitive-behavioral therapy, conversation analysis, RCT, mirroring, reformulating, psychotherapy process research

## Abstract

The conversational actions of reformulating and mirroring constitute some of the core intervention techniques of psychotherapy. The purpose of the present study was to investigate the way in which therapists in cognitive-behavioral (CBT) and psychodynamic therapy (PDT) use reformulating and mirroring strategies to return patients’ prior talk and how their differential usage can be viewed in light of the respective manualized recommendations. A mixed methods approach was applied using qualitative data that derived from a RCT. The data collection consisted of 200 excerpts assembled from both treatment conditions. The method of Conversation Analysis was used to determine the practices that accomplished instances of reformulating and mirroring, and to examine their distinct implications for subsequent talk. The quantitative analysis revealed that cognitive-behavioral therapists are significantly more likely to use reformulations, which is in harmony with what is suggested in CBT’s treatment manuals. Psychodynamic therapists’ frequent use of transformative formulations is, by contrast, unexpected in regard to the suggestions of the treatment protocol, as these interventions steer toward topical closure. Compared to the CBT condition, psychodynamic therapists were still significantly more likely to rely on mirroring strategies, which *are* in line with PDT’s theoretical preference. Our findings raise the question whether alleged differences in treatment styles, as they are imposed by RCT methodology, are actually tangible in manual-guided clinical practice.

## Introduction

The use of manual-based therapy treatment implies an orientation toward manual-informed conversational behavior. It asks for adherence to static and generic guidelines and strategies in an environment that, by nature, demands interactional flexibility shaped by the patient’s individual needs. Standardization of treatment goes hand in hand with randomized controlled trial methodology but carries the risk of becoming an “operational straitjacket” (Wolberg, 2013, p. 922) and is therefore often discussed in terms of its transferability to and utility for clinical practice (e.g. Wilson, 1998; Westen et al., 2004; Vanheule, 2009). Surprisingly little attention is devoted to the question whether treatment manuals actually translate into concrete and observable conversational practices. The aim of the present paper is to examine how, from a conversation-analytic perspective, therapists’ second versions of prior patient talk are deployed differentially across cognitive-behavioral therapy (CBT) and psychodynamic therapy (PDT) and how this can be interpreted in view of the respective treatment manual.

In this paper, we will discuss two types of conversational second versions, with which therapists return prior utterances to their patient. First, we will discuss the conversation-analytic notion of reformulating, which refers to second versions of prior talk that entail lexico-syntactic transformation. Second, we will reflect on Ferrara’s (1994) notion of *mirroring*, in which therapists provide second version by selecting and repeating “a key portion of the client’s utterance” (p. 119). Third, we will analyze data from a randomized controlled trial by using a research method with a particular sense for detail, the method of conversation analysis (CA), to study the differences in therapists’ second versions in CBT and PDT. Lastly, we aim to determine how these differences relate to the suggestions outlined in the respective treatment manuals. Our aim is to investigate whether the use of conversational practices that provide second versions of preceding patient talk is indeed guided by therapy style and if so, what sequential consequences these different practices have.

The CA-coined notion of reformulating refers to a reproduction of preceding talk that inheres and reflects the second speaker’s understanding of prior utterances. As the content of these prior utterances becomes transformed, or *locally edited* (Antaki, 2008), the second speaker not merely rephrases what has been said but also negotiates what is relevant to the current stretch of talk. In therapeutic encounters, formulations serve as an interactional vehicle for a multitude of actions (Voutilainen and Peräkylä, 2014), such as providing a summary of the aforementioned, displaying and checking one’s understanding, zooming in on or filtering out specific aspects of a narrative or facilitating its more detailed exploration. Just as this is key to the practice of a therapist, reformulating also is a valuable means of insight for the patient. After all, the therapist’s formulations provide a mirror reflecting what the patient has been presented so far and what he himself/she herself has made relevant, or as Ferrara (1994) puts it: “if insight is the ability to see inside, then therapist’s formulations are models of insight for clients” (p. 111).

Garfinkel and Sacks (1970) introduced formulations as a practice speakers deploy for meta-communicative descriptions of conversational sequences or achievements. In that sense, the term *formulating* referred to all occasions in which reflexive commentary made prior parts of the conversation become the topic of the conversation. Heritage and Watson (1979) redefined this concept and laid the groundwork for future research on formulations. According to them, formulations “characterize states of affairs already described or negotiated (in whole or in part) in the preceding talk” (p. 126). Three essential properties of formulating were identified: (1) a formulation preserves relevant aspects of prior talk, (2) deletes certain other aspects and (3) transforms the selected material (Heritage and Watson, 1979). The concept thus shifted from Garfinkel and Sack’s initial observation of a practice that reviews preceding talk on metalevel to a practice that reproduces and transforms a prior speaker’s utterances (Childs, 2015). This restricted the concept to a speaker proposing an inference, i.e. *upshot*, or a description of the overall *gist* of prior talk by another participant (Heritage and Watson, 1979). Reformulating facilitates a change from detailed and complex patient narratives into shortened and seemingly more precise subsequent versions. In this way, formulations do fixative work as larger conversational undertakings become segmentalized into confirmable chunks of talk, which render the content preservable and reportable (Heritage and Watson, 1979). Further, therapists’ formulations may close down certain avenues of talk and transform patients’ symptoms or experiences into a suitable shape for diagnosis and history-taking (Antaki, 2008).

A great deal of conversation analytic research has focused on formulations in institutional settings, but especially the use of therapists’ formulations in psychotherapeutic encounters is well-researched (e.g. Davis, 1986; Drew, 2003; Antaki et al., 2005; Antaki, 2008; Weiste and Peräkylä, 2013). Antaki et al. (2005) even accredit formulations “the royal road into the practices of psychotherapy (p. 629f.)”. However, they also claim that the concept has become a fairly permissive one due to its potential to encompass any retrospective description of events described or implied earlier on (Antaki et al., 2005). This demonstrates that determining specific practices that accomplish formulations – and how the social context in which they are situated shapes them – is a challenging affair. Reformulating practices are not necessarily equipped with a fixed set of indicators such as inference markers but comprise various forms of reproductions. These can be detected as such by investigation of adjacent utterances in which prior talk is rephrased or (partially) repeated by a second speaker within the larger conversational undertaking (see Method section for further description of the excerpt selection). Reformulating thereby shows some similarities to the technique of interpretation. Conversation analysts, however, distinguish between both kinds of therapist statements as formulations offer a candidate reading without altering the point, sense or gist of the preceding talk, while reinterpretations include the second speaker’s own perspective (cf. Antaki, 2008).

The current study follows the comparative design of the Ghent Psychotherapy Study (GPS) in order to explore how CBT and PDT techniques shape second versions of patients’ prior talk. A similar study is the comparative analysis on cognitive therapy and psychoanalysis conducted by Weiste and Peräkylä (2013). They discovered two types of formulations that were commonly employed in each therapy approach: Formulations that highlight descriptive elements and thereby preserve therapeutically relevant information and formulations that rephrase descriptive elements of the patient’s narrative and thereby propose more of a transformation (Weiste and Peräkylä, 2013). Two other types of formulations were found to be “related to the core tasks of specific psychotherapeutic approaches” (Weiste and Peräkylä, 2013, p. 319): Relocating formulations that link a patient’s description to other experiences and exaggerating formulations that construct a challenging redesign. Weiste and Peräkylä’s categories were primarily based upon the formulations’ interactional function. The present study embarks from the practices that accomplish second versions, i.e. their design-features, and investigates how reformulating has distinct sequential consequences compared to mirroring across CBT and PDT.

Second versions of prior talk also occur in the form of repetitions, previously referred to as *mirroring*, in which elements of prior talk that appear to be “of particular value to pursue” are repeated by the therapist (cf. Ferrara, 1994). According to Ferrara (1994), responding with iterations is *strategic* as it displays attentiveness but also seeks further elaboration on the repeated item(s) of the preceding talk. In this way, repetitions have a double character: they are as much of a retrospective action as they are an anticipatory action, preparing the ground for the following (or more specifically, the extension of a) stretch of talk. Hence, mirroring preceding utterances counts as an indirect request for elaboration that simultaneously determines the focus of the invited response.

Ferrara (1994) further points out that mirroring not only serves the therapist in receiving more therapeutically relevant information about and from the patient. Repetition also has an impact on the patient in that it increases his or her awareness of what has just been put into words (Ferrara, 1994). The patient basically listens to him- or herself, which presents the possibility of considering those words and their meaning more carefully. Ferrara’s considerations are entirely in harmony with some of the fundamental principles of the psychoanalytic technique. In his elaboration on the punctuation of patients’ discourse, Fink (2007) refers to the verbatim repetition of words as a strategy that “sheds new light on them, allowing them to be heard differently” (p. 43). Psychoanalytic interventions such as punctuation are designed to elicit and explore meaning from the patient’s speech that otherwise would remain implicit and omitted (Fink, 2007).

It must be noted here that mirroring, as introduced by Ferrara, is not equivalent to the concept of equivocal interpretations. Repetitions that return preceding talk back to the patient not necessarily draw on ambiguous elements in the patient’s preceding turns. Still, a “hidden” meaning of the patient’s word choice can become uncovered and the therapist certainly opens up the conversation toward a more detailed exploration of aspects in the patient’s narrative that appear to be of importance. Repetition relieves the therapist from having to ask for elaboration in the form of a question or imperative that may introduce new vocabulary. Instead, the therapist maintains *technical neutrality* (cf. Kernberg, 2016), while opening up the conversation toward new material and potentially facilitating a change of perspective by the patient when confronted with his or her very own language. Repetition thereby provides a seemingly affiliative turn that refrains from introducing potentially suggestive terminology into the interaction, which is an exemplary practice in the psychoanalytic treatment style (cf. Fink, 2007, p. 27).

This study attempts to examine the varying practices through which therapists return preceding patient talk to the patient. We analyzed qualitative data from a randomized controlled trial including two treatment conditions: CBT and supportive-expressive PDT. These data allow for a comparative analysis in order to investigate whether formulating or mirroring prevail in the respective treatment conditions. With this approach, we aim at investigating adherence to treatment suggestions and at contributing to our understanding of the way actual practice is informed and shaped by manual guidelines.

## Materials and Methods

### Setting and Procedure

For this study, audio-recorded therapy treatments offered for patients with Major Depressive Disorder (MDD) were selected from a database assembled for the *Ghent Psychotherapy Study* (GPS; Meganck et al., 2017). This large-scale randomized controlled trial compared predominantly explorative (PDT) and predominantly directive therapy (CBT) in the treatment of depression. Each of the therapy treatments, which took place in Flanders, Belgium, consisted of 16–20 sessions involving one patient and one therapist. Each session had a duration of 40–60 min. The sessions were audio recorded. All therapists that participated in this study had 3–8 years of clinical experience had postgraduate training in psychoanalytic therapy or CBT and received additional training in order to conduct specific treatments for the purpose of the GPS (cf. Meganck et al., 2017). The GPS was approved by the Ethical Committee of the University Hospital of Ghent University (Belgium; EC/2015/0085). All participants gave their written informed consent prior to enrolling in the trial.

### Participant Characteristics

The participants were recruited in Flanders, Belgium. Inclusion was based on the current diagnosis of MDD according to the DSM-IV, a minimum score of 14 on the Hamilton Rating Scale for Depression, age between 18 and 65 years old, sufficient knowledge of the Dutch language and dominance of either dependent or self-critical personality characteristics. The GPS excluded patients with a current diagnosis of psychosis, delusions or bipolar disorder, acute suicidal risk, primary diagnosis of substance abuse/dependence, or evidence of a medical condition that prevented full participation in the treatments (see Meganck et al., 2017).

### Treatment

The patients were randomly assigned to one of the treatment conditions: CBT or supportive-expressive PDT. Four clinical psychologists received training about CBT techniques according to the cognitive behavioral protocol for depression by Bockting and Huibers (2011) based on the manual proposed by Beck et al. (1979). Beck et al. (1979) describe CBT as an active, directive, time -limited and structured approach during which the patient learns how to identify and modify faulty thinking and dysfunctional behavior – e.g. by Socratic questioning – and to recognize and actively change the cognitive patterns leading toward it (Bockting and Huibers, 2011). By that the therapist and patient are collaboratively engaged in the identification and evaluation of cognitions and build hypotheses around the patient’s thinking and underlying assumptions (Beck et al., 1979). Training sessions were also provided to four clinical psychologists learning about PDT techniques according to the unified psychodynamic protocol for depression (the UPP-depression, Leichsenring and Schauenburg, 2014), which integrates empirically supported psychodynamic interventions for depression. Leichsenring and Schauenburg (2014) propose a short-term, face-to-face therapy during which “the therapist adopts a more active stance than in classical psychoanalysis” (p. 135). In addition to the UPP-depression, the psychodynamic therapists in the GPS were guided by Luborsky’s manual (1984).

### Sampling Procedure

The case selection for this study was conducted in the context of an overarching research project on the interactional practices of psychotherapy and was approached from two points: requirements relating to the general suitability of potential cases from the larger corpus and extreme case sampling. The three general selection requirements were as follows: (1) The data had to be available at the start of this research study; (2) Patients had to give specific informed consent to let the audiotapes of the sessions being used for research purposes; (3) The patients had to have surpassed a minimum of five sessions of the treatment to ensure that the distinct features of the treatment style had been implemented after the establishment of a working alliance between therapist and patient. Cases that met these criteria were selected from the larger corpus. In order to select a comparable amount of cases from each condition, we selected two cases with extreme profiles in anaclitic as well as introjective personality style, respectively, which resulted in a total of eight cases. Personality styles were assessed during face-to-face interviews and subsequent prototype matching by three trained researchers, who rated the interviews individually and then discussed their ratings in order to reach consensus (see the GPS protocol for a detailed description of the baseline assessment; Meganck et al., 2017). The inclusion of the two personality styles was adapted from the study design of the GPS; illness severity did not play a role for the determination of differences in therapist interventions (reformulating versus mirroring).

### Data Analysis

A mixed methods study was conducted as the data analysis was split into two stages. The first stage, the qualitative analysis, was focused on the conversation-analytic investigation of the therapists’ second versions of preceding patient talk in CBT and PDT. In the second stage, the results were then quantified in order to examine the distribution of reformulating vs. mirroring.

In the tradition of CA, the selected phenomenon is at first studied as an isolated, single manifestation (Sidnell, 2013). After that, conversation analysts make use of collections consisting of excerpts in which the phenomenon occurs. In doing so, its recurrent, generic properties can come to light (Sidnell, 2013). Hence, CA research finds evidence from collection studies, like the current one. In order to identify the various reformulating strategies in our dataset, we assembled a collection of excerpts that consisted of any sequence in which reformulating was done, starting from session 6 onward. We applied the same procedure for excerpts in which the therapist employed mirroring, i.e. repetition of prior talk. We transcribed the excerpts using the Jeffersonian notation (see Hepburn and Bolden, 2017).

Our analysis by means of CA resulted in five subcollections. These differed with respect to the different practices that accomplished the formulations and repetitions. The process of data collection and analysis was carried out by the first and second author or during data sessions with the first and sixth author present.^1^ During the initial analysis of 228 excerpts selected from the four cases with anaclitic personality style (50 sessions in total), no differences in the distribution of formulations or repetitions between the beginning, middle and end phase of the treatment were found. The selection of excerpts from the remaining four introjective cases was limited to the first 25 in the treatment sessions 6–20. Restricting the excerpt selection ensured that the qualitative analysis remained feasible and could be carried out in a thorough manner, but also ensured that possible other practices could still be identified.

For the second stage of the data analysis, the quantitative analysis, the first 25 excerpts of all eight cases were selected, which resulted in a collection of 200 excerpts. A Binomial test and Fisher’s exact test were used to compare the frequency of the formulations vs. repetitions across the two treatment conditions. These findings alongside with the conversation-analytic findings from our collection study were then compared to the concepts outlined in the literature as well as the therapeutic modalities outlined in the manuals.

### Conversation Analysis

We analyzed our data using the method of CA. CA is a qualitative research method developed in the 1960s–1970s by Harvey Sacks, Emanuel Schegloff, and Gail Jefferson (Pomerantz and Fehr, 2011). It originally set out to study the structure and organization of everyday social interaction, but its scope quickly expanded into the exploration of all kinds of spoken discourse including institutional interaction. CA embraces that the dynamics of interaction heavily rely on the participant’s consistent orientation toward the exchange and management of turn of talk. Conversation analysts study this interactional behavior in close detail, how it reflects peoples’ understanding of each other’s action in talk and the way social relations evolve alongside with it. This analytic quality emerges from CA’s agnostic perspective on participants’ knowledge, status and relationships, or underlying motivations. Instead, it focuses exclusively on observable utterances, patterns and (ir-)regularities in naturally occurring conversational data. From the observation and analysis of the gradually unfolding talk in interaction, conversation analysts then draw inferences about how we understand and construct our social world.

In the same vein, applying CA to psychotherapy lets us catch a glimpse of the strategies through which *doing* psychotherapy is actually accomplished (Westerman, 2011). Since CA first migrated into the realm of psychotherapy research, e.g. with the oft-cited 1986 study by Davis, a number of different actions and communication patterns relevant to the therapeutic process were investigated (for an overview see Peräkylä et al., 2008; Peräkylä, 2012). This concerned matters such as alignment between therapist and patient (Muntigl et al., 2012), the management of resistance patients conceal in their uptakes (Vehviläinen, 2008), or even the way patients’ descriptions of dreams enable affect recognition (Peräkylä, 2008). As the microanalysis of transcribed data takes every interactional detail into consideration, CA attends so subtle or hidden movements, or “micro-signals” (Streeck, 2008), and therefore disposes of a sensitivity that may inform us of conversational strategies, which contribute toward successful treatment (Madill et al., 2001).

Naturally, speakers in psychotherapy show the same orientation toward compliance – in CA this is referred to as the concept of preference organization – as in everyday interaction. However, the therapeutic dialogue does not follow some of the social norms we are accustomed to. Reluctance to talk about intimate thoughts and personal experiences, for example, may become concealed by hesitation markers or delay, i.e. features that are hardly noticeable and can become interpreted as part of conventional conversational behavior. One advantage of using CA is that it has a particular sensibility at its disposal that allows the researcher to detect such discrepancies in talk. Within this present study, CA therefore serves as the microscope through which we aim to unravel therapists’ reformulating and mirroring strategies and their compliance with the respective treatment manuals.

## Results

The therapists made use of five strategies to compose second versions of prior patient talk. Three practices were identified that accomplished formulations and two practices that accomplished mirroring. A total of 147 formulations was found (73.5%), 86 in CBT and 61 in PDT. The data further contained 45 instances of mirroring (22.5%), 11 came from CBT and 34 from PDT sessions.

### Formulations

Our data show that therapists from both schools of psychotherapy primarily make use of formulations that build on prior utterances and use them as a semantic resource. Formulations entail a transformation of prior talk and convert preceding turns into a different format. This transformation of selected elements elevates the content to a more specified or elaborated second version. At the same time, these formulations contribute to the progressivity of the talk as they enable the participants to retrospectively negotiate aspects that are of mutual relevance and allow the therapist to steer the talk into therapeutically meaningful directions.

The first reformulating practice we found in our data consisted of marked formulations, which are marked by the discourse marker *so* or include the subjectivity marker *actually* and typically present a description that summarizes preceding turns by the patient (see Excerpt 1). The second type of reformulating practice concerns formulations that start with the discourse markers *but* or *and* (see Excerpt 2). These formulations typically address contrasting or locally missing elements that were mentioned earlier on and thereby initiate some form of extension. Formulations that specify relevant, individual entities of patient talk compose the third type of reformulating practice (see Excerpt 3). These specifications implement the highest degree of transformation and propose relevant expressions in an *ad hoc* manner, allowing the therapist to introduce some interpretation.

Excerpt 1 shows a prototypical example of a formulation (see Table 1). The patient (P) is a member of a religious organization and explains that she is known to be deeply religious despite the fact that she does not express her beliefs outwardly through her appearance (lines 5 and 6). The therapist (T) reformulates the gist of the preceding account into “so it is what is on the inside that counts” (line 11). This *so*-prefaced formulation displays understanding and reformulates the patient’s words in a refined yet generalized manner.

**TABLE 1:** Marked formulation (PDT).

01	P:	en ↑toch zijn d’r een aantal, .hh
		*and yet there are a couple of [people]*
02		die eh binnen [naam organisatie] ook denken,
		*who uh within [name organisation] who also believe*
03		(0.4) eh ik heb daar geen discussie mee hoor of zo eh
		*uh I am not arguing with them [prt] or something uh*
04		maar die e- z- eh even lang als mij bekeerd zijn,
		*but who e- z- uh have been converted just as long as I am*
05		en die ↑heel goed weten hoe diep gelovig da ’k ben
		*and who know very well how deeply religious I am*
06		ook al ben ik het uiterlijk niet
		*although I do not show that in my outer appearance*
07		[en ben ik.hh
		*and I am*
08	T:	[mh ↑mh
09	P:	die heel goed weten wa- hoe waar dat bij mij zit.
		*who know very well how honest my beliefs are*
10		(1.2)
11	T:	**dus hetgeen dat van binnen zit dat ↑telt**
		***so it is what is on the inside that counts***
12	P:	voilà ik vind da ook (0.9) ja.
		*voilà I believe that, too yes*

In lines 1–10, the patient produces a detailed account about the veracity of her belief. This account includes the reference to people within the organization (lines 1 and 2), with whom she is not arguing (line 3), but who have been converted for a similar amount of time (line 4) and who know very well how honest her beliefs are (lines 5 and 9). The inclusion of all these various attributions makes it a rather complex and imprecise description. With the acknowledgment token in line 8 the therapist demonstrates listenership. After a short silence in line 10, he then transforms the lengthy and detailed multi-unit turn of the patient into a more abstract and condensed second version (line 11), which excludes information about other members and focuses on the role religion has for the patient (i.e. something that is present “on the inside” and does not need to be shown off externally). The formulation moves the topic – the veracity of her belief – into (pre-)closing, leaving it to the patient whether to accept (preferred response) or to reject the formulation (dispreferred response), and accordingly whether to close the topic or to extend it by correcting or adjusting the formulation. The positively formulated declarative conveys the preference for agreement, that is acceptance in the patient’s response, in order to let this interactional project succeed (Mazeland, 2006). The patient shows strong agreement in line 12 (“voila”). Her response further indicates approval of an assessment (“I think so, too”), which implies that she understood the formulation as an affiliative action by the therapist. After a short pause she ends her turn with the confirmation token “yes,” reinforcing her acceptance of the therapist’s formulation. The excerpt demonstrates how marked formulations propose summarizing descriptions of preceding turns and have the potential to move the respective (sub-)topic toward closure.

Excerpt 2 shows an example of a formulation prefaced by the discourse marker *but* (see Table 2). At the beginning of this excerpt (lines 1–5), the therapist reflects on the patient’s behavior in the company of others and proposes “cutting yourself off” (line 3) as a typical reaction. The patient shows some resistance toward this term (line 7) and explains in a lengthy response that “cutting himself off” is not what he experiences in these moments (lines 10–22). In lines 24–28, the therapist provides a marked formulation prefaced by the turn-initial discourse marker *but*.

**TABLE 2:** Marked formulation (CBT).

1	T:	en dan merk je van (0.3) dat je
		*and then you notice [prt] that you*
2		(2.3) dat jouw reactie daarop is
		*that your reaction to that is*
3		(0.8) e:hm misschien wat (1.4) juist weer wat afsluiten (.)
		*uhm maybe somewhat cutting [yourself] off again*
4		e:hm en zeggen van a:ah pf
		*uhm and to say like oh [well]*
5		(0.8) maakt toch allemaal niet uit,
		*all that does not matter anyways*
6		(2.0)
7	P:	ja pf maar ’t is (1.0) ja denk afsluiten,
		*yeah [well] but it’s yeah I believe cutting off*
8		(0.5)
9	T:	of is dat anders?
		*or is that different*
10	P:	ehm ’t is wel anders dan ge[lijk da da ’k zou zeggen van
		*uhm it is certainly different than if I would say like*
11	T:	[↑okay
12	P:	(0.8) eh ’t is nie echt in uzelf keren of zo
		*uh it is not really going into yourself or so*
13		(0.6)
14	T:	[okay
15	P:	[’t is ↑meer (.) mij distantiëren van da ’s
		*it is more like dissociating myself from*
16		(1.1) soort mensen of ai ja
		*that kind of people or [prt] yeah*
		[lines 17-21 omitted]
22		de stommiteiten van eh sommigen ((*grinniken*))
		*the stupidity of certain people ((chuckling))*
23		(0.6)
24	T:	okay (0.2) okay (0.3) **maar dat is iets anders als als**
		*okay okay* ***but that is different to***
25		**eh je afslui[ten of dingen niet meer**
		***uh cutting yourself off or***
26	P:	[ja ja ja
		*yes yes yes*
27		(0.7)
28	T:	**dingen niet meer ervaren** (0.4) okay
		***not experiencing things anymore okay***

In his lengthy response (lines 10–22), the patient states that he is not cutting himself off, but that he is rather dissociating himself from the stupidity of certain people. The therapist demonstrates understanding with the repetitive use of the response token “okay” at the beginning of line 24. He then proposes a pre-closing formulation by restating that the patient’s behavior is different to “cutting yourself off or not experiencing things anymore,” prefacing the reconstruction with *but*. This turn-initial discourse marker naturally carries a contrastive stance (Mazeland and Huiskes, 2001). The contrast lies in the organization of turns, i.e. the arrangement of lines of talk, and does not mean that the participants are in substantial disagreement (Mazeland and Huiskes, 2001). Clearly, the therapist is not arguing against the patient’s description of his own experience. Rather, the formulation is doing repair work that indicates the inaccuracy of the initially proposed term and therefore seems to be used for the purpose of clarification. Since the patient’s confirmation in line 27 comes in quite early in the turn, the formulation – and the subsequent use of yet another “okay” in line 28 – closes this line of talk.

A different kind of transformative formulation we encountered in our data was specifications. These are employed in more of an *ad hoc* manner but nevertheless demonstrate listenership and display engagement. Specifications usually introduce keywords into the interaction that have the potential to reoccur throughout the session or even entire treatment. In that sense specifications provide new vocabulary that may have been unavailable to the patient. However, they can retain an almost commentative status depending on the uptake by the patient. Excerpt 3 shows an example of a specification (see Table 3). The patient is sharing an anecdote about the way her mother dealt with spiders (lines 1–10), which is then reformulated by the therapist into “though lady” in line 12. This formulation focuses on a general character trait of the mother, omitting details about specific events and introducing an abstract version that mirrors her rigorous conduct.

**TABLE 3:** Specification (PDT).

1	P:	mijn moeder had ook geen moeite met ↑spinnen he,
		*my mother did not struggle with spiders [prt]*
2		(0.9)
3	T:	nee? (0.4) [gij wel?
		*no? but you did?*
4	P:	[(nee) die die pakt-
		*(no) she she grabs*
5		eh- ik ben d’r niet te gek van
		*uh- I am not too fond of them*
6		(0.7)
7	T:	[(m:h)
8	P:	[ik ga d’r zo met nen slof naar ↑slaan,
		*I hit them with [one of my] slippers*
9		(1.0) of ze buiten jagen,
		*or chase them outside*
10		(0.8) maar mijn moeder die pakte dat ge↑woon voilà
		*but my mother just grabbed it voila*
11		(10.0)
12	T:	**harde tante.**
		***tough lady***
13		(0.6)
14	P:	↑ja ((grinnikend))
		*yeah ((chuckling))*
15		(2.0) beetje harde tante,
		*a bit of a tough lady*
16		(0.6) (he) ik noem mijn ↑eigen moeder een beetje harde tante, (3.0)
		*(he) I call my own mother a bit of a tough lady*
17		(3.0) maar ’k pro↑beer haar te begrijpen
		*but I am trying to understand her*
18	T:	mh ↑mh

In lines 1–10 the patient is establishing a characterization of her mother that contrasts with her own way of dealing with spiders. After a pause of 10 s, the therapist makes the characterization more explicit by proposing “though lady” (line 12). As can be seen in this excerpt, specifications tend to zoom out to a more generalized interpretation and bring details of the patient’s narrative to a higher level of abstraction. The therapist’s formulation is not just concerned with the way the patient’s mother dealt with spiders, but characterizes as well as assesses her as a tough lady in general. It is notable that the formulation is proposed after a remarkably long silence (see line 11) but immediately solicits a new stretch of talk from the patient (lines 14–17), starting with a confirmation and a somewhat downgraded second assessment [“yeah (2.0) a bit of a tough one”] in line 15. Specifications provide emergent keywords and transform prior talk into categorical entities. Depending on the patient’s uptake, specifications function in a more complementary way or even as a repair in case of unavailability of relevant vocabulary. This practice is similar to the so-called *notionalizations* introduced by Deppermann (2011), as both practices accomplish a selective focus, transforming lengthy accounts with temporal and personal details into more specified and generalized attributions or categories. Inferences are not as explicitly drawn (through the omission of discourse markers) and topics are not as deliberately closed as with marked formulations.

The conformity of the abovementioned transformative practices with the three essential properties of formulating as proposed by Heritage and Watson (1979) is most straightforward. In the abovementioned types of marked formulations an in specifications, a high degree of transformation is introduced as therapists translate preceding utterances into their own words while keeping a strong orientation toward the aforementioned. Especially marked formulations have the potential to move topics into (pre-)closing, thereby capturing an *outcome* to a previous stretch of talk. The formulation renders this outcome preservable in concrete terms. The patient gets confronted with new terminology, which might bring to the surface what he sought to express but with which he may also have different associations. The same is valid for specifications. This suggests that with formulations, i.e. marked formulations and specifications, the therapist and patients are working toward a *mutual* understanding that is very much created in a joint effort rather than solely by the patient himself.

### Mirroring

Apart from second versions of preceding talk that were formulated in a transformative manner, therapists also offered second versions in a non-transformative way, i.e. in the form of repetitions. However, these instances of mirroring were in our data much less frequent than formulations: we only found 45 instances. We identified two practices that accomplished mirroring: selective citations and quotative expressions. The practice of selective citations is identical to what Ferrara (1994) describes as a typical property of mirroring: “a salient phrase (noun phrase, verb phrase, prepositional phrase), not a clause, is picked up from the preceding discourse and uttered with downward intonation” (p. 119). Excerpt 4 shows an example of a selective citation (see Table 4). The patient is talking about her experiences as a child and the relationship with her mother. In line 16 the therapist repeats the word “pity,” which was previously introduced by the patient in line 11.

**TABLE 4:** Selective citation (PDT).

1	T:	is ’t dat je v- voelt u dat kind?
		*is it that you f- do you feel that child*
2		(0.7) of is ’t dat je nu dat kind beter begrijpt?
		*or is it that you can now understand that child better*
3		(0.6) dat je nu begrijpt van wat dat er toen gebeurden
		that now you understand what happened back then
4	P:	((snikt)) da ik e- ik ↑voel dat kind
		*((sniffs)) that I e- I feel that child*
5		ik begrijp het ook (0.9) ik voel de pijn van dat kind
		*I also understand it I feel the pain of that child*
6		(3.5) ((snikt))
		*((sniffs))*
7		zo graag een beetje mama’s aandacht zou willen
		*longing so much for a bit of mother’s attention*
8		(0.5)
9	T:	mh ↑mh
10		(19.7)
11	P:	krijg zo medelijden met dat kind
		*[start to] feel pity for that child*
12		[phh
13	T:	[mh ↑mh
14		((P ademt uit))
		*((P breaths out))*
15		(14.4)
16	T:	**medelij[den.**
		***pity***
17	P:	[en nu nu nu zie ik mijn moeder voor mij
		*and now now now I see my mother in front of me*
18		e- of ik sta daar naar haar te kijken waarom, waarom?
		*e- or I am standing there looking at her why why*
19	T:	mh ↑mh
20		(2.4)
21	P:	ik probeer haar te begrijpen
		*I am trying to understand her*
22		(2.8) want ze heeft ’t ook nie altijd makkelijk gehad
		*because it was not always easy for her, too*

In line 1 the therapist asks whether the patient is able to remember how she felt as a child and whether she can now better understand the events that happened (lines 1–3). In her response in line 4–7 the patient confirms that she again feels the pain of the child that longs for the mother’s attention and that she starts to feel pity for the child (line 11). After the therapist’s acknowledgment in line 13, there is a silence of 14 s. The therapist then repeats the word “pity” (line 16). This selective citation primarily works as an indirect request for elaboration on the term “pity” (cf. Ferrara, 1994). However, the therapist’s turn overlaps with the patient’s next utterances, in which she claims to see her mother again (line 17) and repeatedly uses the question word *why* as if she was asking her mother (line 18). After the therapist’s acknowledgment in line 19, the patient adds that she is trying to understand her mother, knowing that it has not always been easy for her (lines 21 and 22). In this case, the patient does not respond to the therapist’s turn but overlaps and starts a new stretch of talk. With selective citations as the one in Excerpt 4, therapists give particular relevance to one selected aspect of preceding turns. Local repetitions thereby indirectly elicit expansion and project elaboration in the responding turn.

Quotative expressions are a more explicit form of requests for elaboration. The repetition is prefaced by turn-initial indicators such as (*but/and*) *you say* followed by the (partial) repetition of an earlier utterance by the patient. In Excerpt 5, the therapist and patient are discussing the fact that the men in the patient’s life had totally different personalities than her father (see Table 5). The therapist mirrors, i.e. repeats, a key portion of the patient’s utterance in order to expand on the quoted element “insight.” The *and you say-*prefaced repetition was first mentioned by the patient in line 8.

**TABLE 5:** Quotative expression (PDT).

1	T:	[verbaast dat u?
		*are you surprised by that*
2	P:	[en en nu
		*and and now*
3		eh (0.7) nu achteraf nee,
		*uh now in hindsight no*
4	T:	mh ↑mh
5	P:	met al hetgeen da ’k al ervaren heb,
		*considering al what I got to know*
6		e- wat ik erover gelezen heb,
		*e- what I read about it*
7		met met (1.0).hh therapieën en toestanden nee,
		*[concerning] therapies and stuff no*
8		(0.5) omda ’k daar meer inzichten in krijg.
		*because I gain more insight from that*
9		(0.5)
10	T:	mh ↑mh mh ↑mh
11		(2.9)
12	P:	nee dat verbaast mij niet
		*no that does not surprise me*
13		(1.5)
14	T:	**en je zegt van e- inzichten-**
		***and you say [like] e- insight***
15		(3.8)
16	P:	da ’s zoiets van (0.6) hoe kun je nu anders zijn,
		*that is something like how can you be different now*
17		(1.0) of anders doen dan hetgeen da je altijd gezien hebt?
		*or act differently than what you have always seen*
18		(0.6)
19	T:	mh ↑mh mh ↑mh

In line 1, the therapist asks whether that surprises her. In her response in lines 3–12 the patient explains that she is not surprised about this fact because of what she has read about relationships and therapy. In line 8, she states that she has gained more insight from that. In line 12 the patient then concludes her turn by delivering the fitting response type to the therapist’s question in line 1. After a pause of 1.5 s, the therapist repeats “insight” in a quotative format. This repetition is deployed locally as a request for elaboration – inviting the recipient to provide further information – which is then delivered following on from line 16. When deployed at a greater distance from the original utterance, quotative expressions can also direct the patient back to previous utterances that may be of relevance for the current stretch of talk. By readdressing topics that occurred earlier on in the conversation, this type of mirroring is used as a means of topic management.

The non-transformative practices of selective citations and quotative expressions perform mirroring and primarily serve as requests for elaboration. The therapist opens up the conversation toward a more detailed exploration of aspects in patients’ narratives that appear to be of importance and therapeutically relevant. With these indirect requests for elaboration, therapists further demonstrate a high level of alertness to specific utterances that carry a particular emotional weight and can provide valuable insight, but have the potential to become overruled by the entirety of the material that the patient presents in the ongoing session.

### Frequency of Reformulating and Mirroring in CBT and PDT

This section presents the results of the quantitative comparison of all occurring reformulating and mirroring strategies in the dataset. Our data exhibit a high prevalence of formulations: Marked formulations were the most common type of formulation (43.5%), followed by specifications (29%). CBT shows a strong orientation toward the use of marked formulations (60%). PDT treatment shows a more balanced distribution of reformulating practices and uses marked formulations almost as frequently as specifications (32%). Mirroring occurred far less frequently in our data: Selective citations make up only 12%, similar to quotative expressions (10.5%). Selective citations were slightly more common in PDT (15%) than in CBT (9%). Quotative expressions are distinctively more common in PDT (19%) than in CBT treatment (2%). Table 6 shows the distribution of the reformulating and mirroring strategies across the two psychotherapy approaches.

**TABLE 6:** Distribution of reformulating and mirroring over therapy types.

**Strategies**	**CBT**	**PDT**
Formulations	86%	61%
Mirroring	11%	34%
Other	3%	5%
Total	100	100

The prevalence of formulations was compared to that of mirroring by means of a binomial test. More specifically, we tested whether the proportion of formulations was equal to 50% (i.e. the binominal proportion under the null hypothesis). The *p*-value was obtained as the probability (under the assumed binominal distribution with success probability 0.50) to find the observed percentage of formulations or something that is more extreme toward the alternative. In CBT, formulating was significantly more likely to be used than mirroring (86% versus 11% respectively, *p* < 0.001). Similarly, PDT therapists were more likely to deploy reformulating strategies instead of mirroring strategies (61% versus 34%; *p* = 0.007). A Fisher’s exact test further revealed a significant difference between groups. Formulations were significantly more likely to be deployed in CBT than in PDT, while mirroring was significantly more likely to be used in PDT than in CBT (*X*^2^ = 15.99, *p* < 0.001).

## Discussion

The aim of this current study was to investigate whether therapists’ second versions of preceding patient talk are indeed guided by the respective therapy style and if so, what sequential consequences these different practices have. To accomplish our research objective we used CA, a method that allows researchers to gain insight into highly routinized, yet meaningful practices that contribute to the establishment of the therapeutic dialogue. This study focused on two types of interventions that establish second version of prior patient talk, reformulating and mirroring, and aimed at contributing to the general understanding of differences between psychotherapy approaches. In what follows, we attempt to situate the reformulating and mirroring practices identified in our analysis in the broader theoretical context of the two therapeutic approaches. We therefore refer to the manuals that were followed during the GPS. For the cognitive-behavioral condition, we compare our findings to what is suggested in the treatment manual by Bockting and Huibers (2011); for the psychodynamic condition, we discuss our findings in light of the treatment manual by Luborsky (1984). The conversational actions investigated in this study were not mentioned as such in the treatment manuals, as describing formal characteristics of the specific practices of speaking can be considered less applicable to treatment manuals. This holds in particular as Luborsky (1984) refers to psychoanalytically oriented psychotherapy as a less prescriptive treatment during which “much of what the therapist has to do must be tailor-made for the patient and the occasion” (p. 32). Compared to how manuals provide descriptions of interventions, CA does not aim at describing specific effects interventions might have, but employs descriptive terminology for the exhaustive description of observable and concrete interactional behavior.

Our analysis revealed that psychotherapists in both treatment conditions primarily use transformative language to compose second versions of preceding patient talk (73.5% of all second versions were formulations) and that these transformative formulations commonly occurred in the cognitive-behavioral condition (86%). The predominant use of formulations, especially marked formulations (60%), can be interpreted as supportive of two of the main interventions in CBT: the identification of irrational beliefs and automatic thinking, which in a subsequent step get challenged (cf. Bockting and Huibers, 2011). These procedures are performed in a joint effort as the therapists’ responsive turns locally rework patients’ descriptions using their own input, and thereby steer the content into therapeutically more relevant and fruitful directions. From that, we may argue that transformative formulations indicate alignment with CBT’s principle of the *guided discovery* (see Bockting and Huibers, 2011). Similar to *highlighting formulations*, identified by Weiste and Peräkylä (2013), marked formulations convey listenership, demonstrate understanding and the recognition of the patient’s experiences. Marked formulations entail the therapist’s own input, which makes them suggestive, directive and transformative. Their potential to move topics toward closure supports CBT’s objective of identifying and structuring the patient’s thoughts and experiences, and allows patients to “get a grip” on acute issues, pre-existing problems, dysfunctional beliefs and the interplay between these aspects (cf. Bockting and Huibers, 2011).

As outlined in the manual by Luborsky (1984), interpersonally attuned, substantial and consistent understanding is an integral part of the establishment of a working alliance. We investigated the features and prevalence of one intervention type that is in service of this aim: Mirroring opens up the conversation toward new material that can facilitate a deeper level of understanding. These indirect requests for elaboration (that make up 34% of all second versions in PDT) can be considered a suitable intervention technique for psychodynamic treatment as they align with what Luborsky (1984) describes as “restraint about responding interpretatively” (p. 136) and likewise with the following argument by Fink (2007): “the analyst does well to use the exact same words and expressions as the analysand, as opposed to formulating things in his own terms” (p. 26). Mirroring allows the therapist to format his turn in an impartial and non-suggestive manner while still demonstrating receptiveness (as too little responding actions may let the patient doubt the therapist’s attentiveness). Using mirroring when returning preceding talk to the original speaker thus facilitates an unbiased exploration of and reflection on the patient’s perspective and experiences, which is in line with PDT’s theoretical preference.

Therapists in the psychodynamic condition made use of a wide diversity of practices when formulating second versions of prior talk, transformative formulations being the most dominant one (61%). The most common practice in PDT was specification (32%). Specifications are less invasive to patient talk than marked formulations and only occur as minor interruptions to the patient’s flow of speech but introduce a high level of transformation. They serve as devices that transform patients’ complex, vague and tentative descriptions into indexical categories while simultaneously preparing the ground for topical closure (cf. *notionalizations*, Deppermann, 2011). Such a transformation naturally involves interpretation, which is not entirely in line with Luborsky’s (1984) principle of restraining from interpretive responding actions until a profound level of understanding is attained. However, specifications offer an interpretive statement that is limited in its extensiveness and complexity, as proposed by Luborsky (1984; cf. *Principle 9*, p. 136). Our findings thus suggest that psychodynamic therapists act in most cases as if sufficient understanding is already achieved or as if they prefer to introduce their own, and thus transformative, language. By that, therapists are however treading on thin ice: Should the patient disagree with the proposed formulation, this would hint at a potential misunderstanding and the working alliance may be put at risk. In sum, we can conclude that the use of transformative language is suggestive yet commonly deployed in PDT. This finding supports that the transformative nature (as suggested by Heritage and Watson, 1979) is inherent to the reformulating strategies speakers apply in psychotherapy.

In the end, it is not the manual that creates the interaction, nor can the manual be hold accountable for specific features of talk or differences in the way therapists from distinct approaches are speaking; it is both the therapist and patient that locally achieve and co-construct the interaction and shape it into – in a categorical sense – a therapy interaction. It is these participants’ orientation toward distinct norms and structures that establishes a dialogue recognizable as a therapeutic one, which differentiates itself from conversations in other (institutional) settings (cf. Mondada, 1998). In their interactional moves therapists may show an orientation toward actions and practices of speaking that can be seen as congruent with certain approaches or schools, still it is the therapist’s interpretation and locally determined application of those theoretical ideas. In this way, approach-specific training may roughly sculpt the interactional routine, or stream of actions, that becomes deployed and revisited during therapy sessions and throughout the treatment. However, it is especially *in retrospect* that interventions can be put into various approach-specific categories. The therapist’s focus and attention during the session will lie on the conversation he has with his conversational partner, the patient in front of him, and to make this conversation as productive, curing and problem solving as possible. In that very moment, the manual hidden behind him in the back of his bookrack will have much less of a direct impact on the construction of his interventions. As already proposed by Norcross and Wampold (2018), it is therapist flexibility to unique patient characteristics and preferences that starts to prevail in clinical practice (p. 1901).

What we hope to have demonstrated is that RCT methodology and, by extension, manualization imposes a distinctiveness on therapy types that does not necessarily come to light when conversational behavior is investigated inductively. However, these findings rest on the analysis of two types of intervention, reformulating and mirroring. Further research into the conversational practices that accomplish other types of therapy interventions should continue this line of inquiry.

In the following, we address the potential limitations of this study and share our views on them. CA research is generally found to be fairly reliable due to its data-driven approach. However, the methodology of our study is by no means immune to the confounding factors commonly associated with research studies. One factor that occurs to us as potentially confounding concerns the selection of cases and individuals that participated in these interactions. In this study, we included eight cases, and thus eight combinations of therapists and patients. We trust that this selection is broad enough to overrule particularities of an individual’s language use. A follow up study would, however, benefit from a larger sample size in order to investigate whether the same distribution of practices occurs across a more diverse range of cases. We also believe that the Flemish language use in our data is not a limiting factor to the generic properties of the practices we identified. As our analysis was limited to the therapists’ second versions, the responding actions may constitute an interesting topic for future studies using CA.

Previous research by Weiste and Peräkylä (2013) addressed that “research results based on data obtained from one specific psychotherapy approach cannot, without problems, be generalized to and be considered representative of psychotherapy as a whole” (p. 319). With this current study, we can support this assumption. Our findings indicate that conceptual differences between PDT and CBT could certainly be associated with the conversational practices that produce therapists’ second versions. We claim that the reformulating practices of CBT align with its theoretical perspective. Psychodynamic therapists, however, appear to – at least partly – diverge from the manual’s suggestions. This study has therefore demonstrated that, in the case of PDT, there is a gap between theory and actual practice at the level of the use of mirroring. This gap is addressed in Peräkylä and Vehviläinen’s (2003) work on “stocks of interactional knowledge,” that proposes that a dialogue between CA researchers and professionals can enrich the concepts and theories, i.e. the knowledge base, of certain professions. As for the profession in which this current study is situated, we believe that the discussion of conversation-analytic findings during training events for psychotherapists may not only raise awareness about the interactional impact of interventions on the ongoing treatment session, but that it would also create the opportunity to reflect on the suitability of particular manuals and possible suggestions for their improvement.

## Author’s Note

The data for this research was collected as part of the Ghent Psychotherapy Study (Meganck et al., 2017), a randomized controlled trial on the treatment of major depression (registered on the Open Science Framework; ISRCTN 17130982). Our sample consisted of a small subsample of audio-recordings and was selected without consideration of outcome measures.

## Data Availability Statement

The datasets generated for this study are available on request to the corresponding author.

## Ethics Statement

The Ghent Psychotherapy Study was approved by the Ethical Committee of the University Hospital of Ghent University. All participants provided their written consent to participate in this study.

## Author Contributions

AK: main author of the manuscript, main investigator responsible for data-analysis, and interpretation. MH: main reviewer of the manuscript, contributed to the study design, performed the data-analysis, and contributed to the interpretation of the results. TK: reviewer of the manuscript, contributed to the study design, and to the data-analysis and interpretation. RM: reviewer of the manuscript. TL: performed the statistical analysis. MD: reviewer of manuscript, contributed to the development of the study design, and to the data-analysis and interpretation.

## Conflict of Interest

The authors declare that the research was conducted in the absence of any commercial or financial relationships that could be construed as a potential conflict of interest.

## Footnotes

1 Researcher allegiance effects were not examined since the authors that were primarily responsible for the data analysis did not have a background in psychology or psychotherapy. AK and MH were therefore not associated with either of the included psychotherapy modalities and held a neutral stance during the process of data analysis.
